# Gender Effect on Views of Wisdom and Wisdom Levels

**DOI:** 10.3389/fpsyg.2021.725736

**Published:** 2021-10-26

**Authors:** Mimi Xiong, Fengyan Wang

**Affiliations:** ^1^School of Psychology, Nanjing Normal University, Nanjing, China; ^2^Institute of Moral Education Research, Nanjing Normal University, Nanjing, China

**Keywords:** wisdom, gender effect, views of wisdom, wisdom levels, psychological gender

## Abstract

Gender differences in wisdom are an important theme in mythology, philosophy, psychology, and daily life. Based on the existing psychological research, consensus and dispute exist between the two genders on the views of wisdom and in the levels of wisdom. In terms of the views of wisdom, the way men and women view wisdom is highly similar, and from the perspectives of both ordinary people and professional researchers of wisdom psychology, wise men and women are extremely similar. Regarding wisdom level, research has revealed that, although significant gender effects exist in the level of overall wisdom, reflective and affective dimension, and interpersonal conflict coping styles, the effect sizes were small, which indicated that these gender differences were not obvious. It would be desirable for future research to combine multiple wisdom measurements, strengthen research on the psychological gender effect of wisdom, and focus on the moderating role of age on the relationship between wisdom and gender.

## Introduction

Are there gender differences in wisdom? The human debate on this issue has continued for thousands of years. Considering ancient Chinese and Western philosophical comparisons of wisdom between men and women, although there are views that “men and women complement each other,” the idea of “strong men and weak women” always dominates. Regarding this issue, arguably, the most famous classic expressions in the West emanated from the works of Plato. Although Plato admitted that “the best policy for a city is to make women share with men in everything,” (Plato, 1902/2009, p. 313), he also proposed that “doubtless man is superior, as the whole, in capacity and strength” (p. 283). A similar statement in Plato’s Dialogues by Timaeus states that “men who were cowards or led unrighteous lives may be supposed to have changed into the nature of women in the second generation” (Plato, 1892, p. 513). Here, “righteous” refers to conquered fear, anger, and the opposite affections (p. 358). From these remarks, seen through Plato’s eyes, it appears that he believed women had a much lower virtue than men. Later, Christianity inherited this Greek philosophical view of the relationship between wisdom and gender that women are unwise and, therefore, must obey the teaching of their husbands. This can be seen in the Old Testament story of Adam and Eve: Eve lacked self-control and was tempted to steal the forbidden fruit, leading to the expulsion of humankind from the Garden of Eden. Another punishment from Jehovah for her was that “you will adore your husband, and your husband will rule you.”

In Chinese culture, this view is represented by Confucian culture. Confucianism, as a traditional doctrine of a patriarchal society, emphasized the roles and ethical obligations of women, advocated that men are superior to women, and believed that women do not have independent personality and wisdom (Yao, 2000, pp. 183–184). From the perspective of Confucianism, the ideal personality of women is one based on attachment and obedience (Zhu, 2006, pp. 93–127), and their wisdom is mainly manifested in assisting their husbands, raising children, serving parents and parental in-laws, and managing family affairs (Wang, 2019, pp. 140–142). Conversely, the ideal personality of men is that of a gentleman, and their wisdom is reflected in dealing with the relationship between heaven and man, others and himself, body, and mind, and subject and object (Wang and Zheng, 2014, pp. 246–251). Essentially, this view holds that ideal men are wiser than ideal women because the number and difficulty of the tasks of men and the range of benefits derived from their behavior are far better than those of women. From these viewpoints, we can clearly see the gender effect from the Confucian view in the notions of strong, wise men and weak women. In summary, although Chinese and Western philosophers lived in different sociocultural backgrounds and historical traditions, they all believed that women were less wise than men.

As an important part of both Chinese and Western philosophies, this view of gender differences in wisdom has exerted an extensive and profound influence on the collective understanding of wisdom and the relationship between men and women. However, is this standpoint reasonable? Does it conform to objective facts? These questions have long plagued people. Since the 1980s, when the notion of life-span development emerged, with research on the wisdom of the elderly and positive psychology (Yang, 2008), wisdom psychology has attracted much attention. Therefore, the psychological connotation of wisdom has become increasingly clear, and various measures and paradigms consistent with the standards of modern psychometrics have also been developed (Kunzmann, 2019; Webster, 2019). Using these tools, scholars can conduct more objective and in-depth empirical discussions on gender similarities and differences in wisdom and thus provide preliminary answers to the above questions. To expand, discussing possible gender differences in wisdom focuses on two issues: (a) the relationship between gender and views of wisdom and (b) gender effects on wisdom levels. Generally, most philosophers pay attention to only the latter question, whereas psychologists attach equal importance to the two, and significant progress has been made in both fields.

Therefore, a systematic review and brief comment on existing research in the field of wisdom psychology will reveal the relationship between wisdom and gender. This not only provides ideas and enlightenment for follow-up research on the gender effects of wisdom but also provides a powerful reference for the formulation of gender-specific public policies, organizational strategies, and behavioral norms in all aspects of life.

Before embarking on this review, we must clarify that although we do not deny that gender and sex are considerably different in concept and characteristics (Cretella et al., 2019), some researchers use these two terms interchangeably and do not distinguish between them in practice, especially while collecting and analyzing data (Helgeson, 2017, p. 30). When collecting data, most of the previous empirical studies that claim to examine the relationship between wisdom and psychological gender required subjects to report biological sex but not psychological gender. In this sense, these studies are merely exploring the association between wisdom and biological sex (Hyde et al., 2019). That said, studies that explored both the psychological gender effect and biological sex effect of wisdom were included in this review.

## Gender and Views of Wisdom

The view of wisdom refers to opinions on wisdom. The relationship between gender and views of wisdom can be divided into two sub-questions. First, (a) are there differences in views of wisdom between men and women – do men and women think the same about wisdom? This question will be referred to as the “gender effect on views of wisdom.” Second, (b) do people have different views of wise men and wise women? That is, in the collective conception, whether wise men and women have similar personality, competence, and morality and will be referred to as the “gender effect on wise nominees.” These two questions are discussed in detail below.

### Gender Effect on Views of Wisdom

Overall, the ways in which men and women view wisdom are highly similar. Specifically, men and women similarly understand the connotations of wisdom. For example, Glück et al. (2012) asked participants to judge the fitness of given adjectives relating to wisdom according to their own understanding of wisdom. The results showed that 90% of the participants thought that wisdom was more closely related to adjectives such as friendly, intelligent, sensitive, honest, and highly intelligent, and there was no significant gender difference.

Meanwhile, men and women have almost identical opinions of the real-life manifestations of wisdom. “Wisdom events” personally experienced by men and women are different in domain and focus but are essentially the same and can be summarized into the following five categories: helping others and contributing to society to strive for common good, achieving and maintaining a satisfactory state of life, deciding life paths, solving problems and challenges at work, and insisting on doing what they think is right when facing diversity (Yang, 2008). For example, men reported wisdom events as “accurately positioning and adjusting their role in the workplace, and pursuing personal growth” and women reported wisdom events as “maintaining a satisfactory relationship between family members”; these may be far apart in content, but they belong to the same category “achieving and maintaining a satisfactory state of life,” which was defined as people actively pursuing their goals and striving to achieve their ideal way of life (Yang, 2008). Spiritual fulfillment, career success, harmony in interpersonal relationships, work–life balance, and realizing their self-worth all fall into this category.

There are two possible explanations for these phenomena. First, the essence of this type of research is group comparison, which considers the similarities and differences between the general views of wisdom collectively held by respective male and female groups. This approach obscures those outlying views on wisdom held by the general public; therefore, insignificant results between the genders are increasingly likely. For example, a few people may nominate those with high levels of Machiavellianism as wise; in the process of statistical analysis, this minority view of wisdom will be neutralized by mainstream ideas and, thus, will not appear in the results. In this case, unless most men and women hold different views of wisdom, there is little possibility of significant gender differences appearing in the results.

Second, this type of research is mostly an intra-cultural comparison. The viewpoints on “What is wisdom” and “What kind of person can be regarded as a wise person” received from the social environment and practical activities by individuals from the same cultural background are very similar, regardless of gender (Wang and Zheng, 2014, pp. 268–276; Grossmann and Kung, 2018; Grossmann et al., 2020). Consequently, they are extremely likely to form highly consistent views of wisdom. Two empirical studies provided evidence for this assumption that people from the same culture possess a high degree of homogeneity in views of wisdom. Wang and Zheng (2014, pp. 285–294) invited 167 Chinese university students to nominate wise persons, and results found that the average mention rate of the top five wise people (i.e., Laozi, Socrates, Confucius, Mencius, and Marx) was 85.12%. In other words, 85.12% of participants regard these five as wise people. Weststrate et al. (2016) conducted a wisdom nomination study in a sample of Westerns and found that among the 303 wisdom nominees, the top 13 wisest people were nominated 169 times, accounting for 56% of the total. Of course, we do not rule out the possibility that persons from the same culture have differing perspectives on wisdom. After all, individual attributes (such as socioeconomic status and educational background) will also influence views of wisdom. We only want to point out that people from the same culture are more likely to develop similar views of wisdom.

In addition, it is also essential to understand the views of wisdom of individuals from different cultures. Previous studies have shown that individuals from different cultures have certain similarities in their views of wisdom; for example, both Chinese and Western concepts of wisdom include elements of virtue and intelligence and advocate the use of wisdom to solve real-life problems (Wang et al., 2021). However, there are also differences in the views of wisdom among individuals from different cultures (see review, Yang and Intezari, 2019); for example, researchers found that Chinese views of wisdom comprised two culturally specific components – the “spirituality of disengagement” and a “positive mindset” (Hu et al., 2018).

### Gender Effect on Wise Nominees

Wise men and women are highly similar, whether through the eyes of ordinary people or professional researchers of wisdom psychology. Specifically, for ordinary people, their descriptions of wise men are considerably similar to those of wise women (König and Glück, 2012). For example, in the research by Glück et al. (2009), participants were first asked to read two pieces of paper; the first one read “Paul, his friends think he is a wise man,” and the second one read “Paula, is considered by her friends to be a wise woman.” Participants were then asked to evaluate the degree of correspondence between these two virtual characters with 80 adjectival words (or phrases), involving many aspects like knowledge and life experience, fluid intelligence, insight, reflection, openness, emotional management, caring for others, and practical skills. Results showed that there was no significant difference between the descriptions of the participant of “Paul” and “Paula,” which indicated that according to ordinary people, wise men resemble wise women in personality traits and abilities.

Also, wisdom psychology researchers view wise men and wise women similarly, which is mainly reflected in the fact that most of these scholars do not distinguish between gender when defining wisdom. In other words, they believe that there is no need to discuss them separately because the same sets of wisdom theories, as well as scales and paradigms, can be used to understand and measure the wisdom of men and women.

In summary, in the eyes of most people, a wise person is androgynous, with both high levels of masculinity and femininity (Orwoll and Achenbaum, 1993; Ardelt, 2009). This seems to contradict the common belief that society holds different expectations of men and women – social expectation theory; however, it does not. Mainline gender expectations held by ordinary society are aimed at ordinary people and are not suitable for wise persons above the social average in all aspects. For example, men are expected to be agentic, while women are communal (Ellemers, 2018; Eagly et al., 2019). This is true for describing ordinary men and women but is not suitable for depicting wise men and women. This is because almost all existing wisdom psychological models advocate that wise person has both high agency and community; they not only pursue the smooth solution of practical problems and the realization of personal goals but also pay attention to maintaining harmonious and stable relationships with others (Jeste and Lee, 2019).

In addition to asking participants to describe wise men and women, wisdom nomination research can illuminate the gender effect of wise nominees. Nowadays, most wisdom nomination research has found that men are more likely than women to be nominated as wise persons (Weststrate et al., 2016). For example, Yang (2008, 2011) invited 200 adults from Taiwan, China, to write down the names of two people they thought were wise; 66 nominees were collected, and most were men (55, 77%). For another example, in a historical wise people nomination research, 209 participants nominated a total of 106 wise people, of which 81 were men (76%) and only 25 were women (Weststrate et al., 2016).

Does this mean that the general public thought that men are wiser than women? The answer is no. In fact, scholars believe that this finding, while common, cannot truly reflect the view of the public of wise men and women. They prefer to explain this phenomenon by the social atmosphere and the different social statuses of the two genders. Specifically, human history has been dominated by the patriarchal system; in most historical periods, men have been in dominant familial and societal positions. Thus, they have had more opportunities to publicly display their wisdom, whereas women have had comparatively fewer opportunities to be seen outside of the family (Weststrate et al., 2016). Consequently, few wisdom stories of women existed to be passed down orally or recorded. Accordingly, when nominating wise persons, there is a scarcity of wise women to choose from; therefore, women are naturally far less likely to be nominated as wise than men.

## Gender and Wisdom Level

The results of current studies have not yet reached a consensus regarding the relationship between wisdom level and gender. To explore this gender effect more comprehensively, this study systematically reviewed the related published empirical studies.

To collect the relevant literature as comprehensively as possible, we searched in PsycINFO, ScienceDirect, Scopus, Web of Science, EBSCO, and ProQuest Dissertation database using “wisdom” as the title and keyword successively. The search field was limited to Social Sciences, and the search direction was limited to Psychology. The retrieval dates ranged from 2000 to 2020, and the databases were searched on November 25, 2020. Additionally, to retrieve more eligible studies, we searched the references from our retrieved articles for other leads. A total of 4873 papers were identified.

**FIGURE 1:**
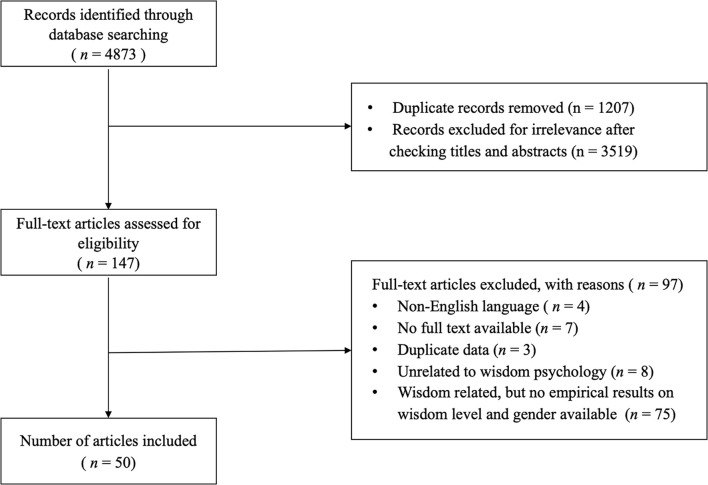
Flow diagram for included studies in the systematic review.

Duplicate documents (*n* = 1207) were deleted first. Subsequently, an initial screening was conducted by reading topics and abstracts; papers that did not target the normal population and were not topically related to wisdom, reviews, and survey reports were deleted (*n* = 3519). Next, a second screening round was performed on the remaining 147 documents. The inclusion criteria were (a) written in English, (b) full text available, and (c) must have reported empirical results of gender effect on the level of overall wisdom or that of wisdom dimensions. Articles unassociated with wisdom psychology and those that were associated with wisdom psychology but did not report any empirical results of gender effects were excluded. Additionally, studies that reported the same sample pool used in previously included studies were also excluded. Consequently, 50 empirical studies met these standards. These two rounds of literature screening processes are shown in Figure 1.

The 50 articles included in this systematic review were coded as follows: study ID, author name, publication year, geographic region, sample size (female ratio), mean age of the sample (age range), wisdom measurement tools, empirical results of the gender effect of level of overall wisdom, and empirical results of the gender effect of level of wisdom dimensions. Two coders coded separately according to the abovementioned inclusion and exclusion criteria, and the coding consistency was 90.9%, indicating that the coding of the literature was effective and accurate. The results after encoding are shown in Tables 1, 2.

**TABLE 1:** Insignificant gender effect on wisdom level.

No.	Author and Year	*N* (Percent of women)	*M*_age_	Measurement	Results of overall wisdom	Results of dimensions
1	Alhosseini and Ferrari, 2019	80 (50.00%)	51.93	3D-WS	N.S.	N.S.
2	Saleem et al., 2017	150 (44.67%)	N/A	WDS	N.S.	N.S.
3	Takahashi and Overton, 2002	136 (50.00%)	57.71	Analytical and synthetic wisdom measures	N.S.	N.S.
4	Ardelt, 2009	642 (73.00%)	46	3D-WS	N.S.	
5	Ardelt and Bruya, 2020	217 (59.50%)	19.5	3D-WS	N.S.	
6	Ardelt and Ferrari, 2019	211 (N/A)	50.63	3D-WS	N.S.	
7	Bang and Montgomery, 2013	639 (N/A)	19.74	3D-WS	N.S.	
8	Beaumont, 2011	320 (68.44%)	20.43	3D-WS	N.S.	
9	Bruya and Ardelt, 2018	308 (60.4%)	19.4	3D-WS	N.S.	
10	Bushlack and Bock, 2018	166 (38.00%)	N/A	BWSS	N.S.	
11	Cheung and Chow, 2019	142 (78.90%)	73.2	SAWS, WDS	N.S.	
12	Dumbravă, 2017	100 (87.00%)	24	3D-WS	N.S.	
13	Glück et al., 2013	170 (52.94%)	57.5	SAWS, 3D-WS, ASTI, BWP	N.S.	
14	Grossmann, 2012	411 (52.40%)	47.16	Other rating	N.S.	
15	König and Glück, 2014	443 (77.70%)	28.2	ASTI	N.S.	
16	Krause and Hayward, 2014	1,154 (64.10%)	63.4	PWS	N.S.	
17	Krause and Hayward, 2015	1,535 (61.00%)	63.3	PWS	N.S.	
18	Le and Levenson, 2005	254 (63.30%)	34.5	ASTI	N.S.	
19	Lee et al., 2019	340 (50.00%)	62	SD-WISE	N.S.	
20	Levenson et al., 2005	341 (72.50%)	34	ASTI	N.S.	
21	Thomas et al., 2019a	524 (51.00%)	58	SD-WISE	N.S.	
22	Webster, 2007	171 (57.31%)	42.77	SAWS	N.S.	
23	Webster et al., 2014	512 (63.67%)	46.46	SAWS	N.S.	
24	Wei and Wang, 2020	487 (27.10%)	25.37	SWIS	N.S.	
25	Wink and Dillon, 2003	201 (53.00%)	N/A	WS	N.S.	
26	Wink and Staudinger, 2016	163 (53%)	73	BWP	N.S.	
27	Krause, 2016	1,535 (60.60%)	63.3	PWS		N.S.

*3D-WS, three-dimension wisdom scale (Ardelt, 2003); ASTI, adult self-transcendence inventory (Levenson et al., 2005); BWP, Berlin wisdom paradigm (Baltes and Staudinger, 2000); BWSS, brief wisdom screening scale (Glück et al., 2013); PWS, practice wisdom scale (Krause, 2016); SAWS, self-assessed wisdom scale (Webster, 2003); SD-WISE, San Diego wisdom scale (Thomas et al., 2019a); SWIS, situated wise reasoning scale (Brienza et al., 2018); WDS, wisdom development scale (Brown and Greene, 2006); WS, wisdom scale (Hartman, 2000).*

*N.S. indicates the result was insignificant. Blank cells indicate either significant results or unreported results.*

**TABLE 2:** Significant gender effect on wisdom level.

No.	Author and Year	*N* (Percent of women)	*M*_age_	Measurement	Results of overall wisdom	Results of dimensions
1	Ardelt and Jeste, 2018	994 (48.80%)	77.3	3D-WS	M < F, *r* = −0.09	Compassionate: M < F, *r* = −0.26
2	Ardelt et al., 2018	14,248 (48.50%)	36.46	3D-WS	M < F, *β* = 0.04	Cognitive: M < F, *β* = 0.04 Compassionate: M < F, *β* = 0.10 Reflective: M > F, *β* = −0.04
3	Brudek and Sekowski, 2019	567 (49.74%)	57.53	3D-WS	M < F	Compassion: M < F, partial *n*^2^ = 0.02
4	Cheraghi et al., 2015	439 (61.73%)	34.09	3D-WS	Interaction effect between gender and age cohorts, partial *n*^2^ = 0.029	Compassionate: M < F, *r* = 0.15 Reflective: M > F, partial *n*^2^ = 0.011 Significant interaction effect between gender and age cohort for all dimensions.
5	Jason et al., 2004	373 (N/A)	24.02	FVS	M > F	Warmth: M > F Intelligence: M > F
6	Kordacová, 2010	167 (85.03%)	20.03	3D-WS	M < F	Affective: M < F
7	Webster et al., 2018	271 (62.36%)	20.37	SAWS	M < F, *r* = 0.13	Reminiscence: M < F, *r* = 0.17
8	Zacher et al., 2013	575 (66.80%)	26.3	3D-WS	Worker: M < F, *r* = 0.16	Student: Affective: M < F, *r* = 0.17 Worker: Cognitive: M < F, *r* = 0.12; Affective: M < F
9	Auer-Spath and Glück, 2019	155 (54.84%)	56.2	BWSS, BWP, MORE	MORE interview: M < F, *r* = −0.228	
10	Brienza et al., 2018	3,962 (55.03%)	32.1	SWIS	Study 8: North American: M < F Profile academic: M > F	
11	Ferrari et al., 2019	189 (62.00%)	N/A	3D-WS	M > F, *r* = −0.17	
12	Gordon and Jordan, 2017	84 (58.33%)	N/A	Other rating	Advice narrative: Main effect: M < F, partial *n*^2^ = 0.02. Interaction effect between age and gender, partial *n*^2^ = 0.01 Moral dilemma narrative: Interaction effect between gender and age, partial *n*^2^ = 0.03	
13	Huynh et al., 2017	623 (65.17%)	29.69	WRS	Study 2: M < F, partial *n*^2^ = 0.01	
14	Krause, 2016	1,535 (60.60%)	63.3	PWS	M > F, *r* = −0.062	
15	Le, 2008	251 (77.00%)	20.2	3D-WS, ASTI	3D-WS: M < F, *β* = 0.14	
16	Pasupathi and Staudinger, 2001	204 (N/A)	N/A	Adopted from BWP	Adolescent: M < F, partial *n*^2^ = 0.03	
17	Verhaeghen, 2019	260 (54.00%)	19.7	ASTI, 3D-WS	ASTI, *r* = 0.05 3D-WS, *r* = 0.13	
18	Webster, 2003	85 (N/A)	52.54	SAWS	M < F, *r* = 0.291	
19	Ardelt, 2009	642 (73.00%)	46	3D-WS		Affective: M < F, partial *n*^2^ = 0.022. Cognitive: interaction effect between gender and age, partial *n*^2^ = 0.012
20	Ardelt and Bruya, 2020	217 (59.50%)	19.5	3D-WS		Time 1: Reflective: M > F, *r* = −0.14; Compassionate: M < F, *r* = 0.14 Time 2: Compassionate: M < F, *r* = 0.16
21	Bang and Zhou, 2014	356 (38.00%)	20.5	Adapted 3D-WS		M < F in nondualistic thinking (*r* = 0.13), non-resentment (*r* = 0.15), and empathy (*r* = 0.17)
22	Beaumont, 2011	320 (68.44%)	20.43	3D-WS		Affective: M < F, partial *n*^2^ = 0.048
23	Bergsma and Ardelt, 2012	7,037 (62.00%)	N/A	3D-WS		Affective: M < F, *r* = 0.12
24	Booker and Dunsmore, 2016	263 (67.30%)	N/A	BWP		M < F in use of procedural knowledge, lifespan perspective, relativism of values, management of uncertainty
25	García-Campayo et al., 2018	624 (75.65%)	44.7	3D-WS		Reflective: M > F, *p* = 0.037, Cohen’s *d* = 0.22 Affective: M < F, *p* = 0.024, Cohen’s *d* = 0.30
26	Grossmann et al., 2012	411 (52.40%)	47.16	Other rating		Japan: M < F in flexibility factor (*r* = −0.18), perspective taking factor (*r* = −0.18), and recognition of limits of knowledge factor (*r* = −0.17)
27	Grossmann and Kross, 2014	Study 1: 104 (64.42%) Study 2: 120 (66.67%)	Study 1: 20.35 Study 2: 19.63	Wise reasoning questions		Study 1: Compromise: M < F, partial *n*^2^ = 0.04; Limits of knowledge: M > F; Perspectives: M > F; Emotional reactivity: M < F Study 2: Limits of knowledge: significant target × gender interaction, partial *n*^2^ = 0.02
28	Moraitou and Efklides, 2012	446 (51.35%)	49.67	WITHAQ		significant in one item (PW 4, Cohen’s *d* < 0.20)

*FVS, The Foundational Value Scale (Jason et al., 2001); MORE, The MORE Interview (Glück et al., 2019); WITHAQ, The Wise Thinking and Acting Questionnaire (Moraitou and Efklides, 2012); WRS, wise reasoning scale (Huynh et al., 2017). Refer to abbreviations given in Table 1.*

*M in the result column indicates male, F compared with M indicates female. Blank cells indicate either insignificant results or unreported results.*

Overall, the current empirical research has generated contradictory results: (a) the wisdom level of men and women is basically the same and (b) significant gender differences exist at the wisdom level.

### Insignificant Gender Effect on Wisdom Level

#### Insignificant Gender Effect on the Levels of Overall Wisdom

Regarding overall wisdom, extensive research varied in theoretical basis and measurement tools and found no significant effect on the level of overall wisdom. Baltes and Staudinger (2000), based on previous studies on expertise and cultural-historical analyses of wisdom and the standpoints of neo-Piagetian and lifespan development psychology, perceived wisdom as an expert knowledge system in the fundamental pragmatics of life and proposed five criteria to evaluate wisdom levels: rich factual and procedural knowledge, lifespan contextualism, relativism of values and life priorities, and recognition and management of uncertainty. Based on this, Wink and Staudinger (2016) investigated the wisdom levels of 163 white people aged 68–77 years and found no significant difference between genders.

Webster (2007) proposed that wisdom is the ability and intention of an individual to apply critical life experiences to promote the optimal development of self and others. He deconstructed wisdom into five dimensions – experience, emotional regulation, reminiscence and reflectiveness, openness, and humor – and subsequently developed the corresponding measurement tool – the self-assessed wisdom scale (SAWS) (Webster, 2003). A study using SAWS in a sample of 142 Chinese elderly found no significant gender difference in the overall wisdom levels (Cheung and Chow, 2019).

Ardelt (2003) conceptualized wisdom as a personality trait integrating three dimensions, namely, cognitive, reflective, and affective, and produced a corresponding three-dimension wisdom scale (3D-WS). These three dimensions of wisdom correspond to those obtained from previous analyses based on the views of wisdom of laypeople (Clayton and Birren, 1980; Sternberg, 1990). Of these dimensions, reflective refers to looking at events and phenomena from different perspectives to develop self-awareness, avoiding blaming others or circumstances for their present situation, accurately perceiving, effectively regulating emotions, and forgiving self and others. Cognitive refers to the ability and willingness of an individual to comprehend the significance and deeper meaning of phenomena and events dialectically and thoroughly, to recognize the unpredictability and uncertainties of human nature and life. Affective means being sympathetic and compassionate to all people. Similarly, empirical research using this scale found no significant difference in the overall wisdom scores between men and women (Ardelt and Ferrari, 2019).

From the perspective of lifelong development, Levenson et al. (2005) proposed that wisdom reflects individuals no longer rely on externals (such as material, social role, achievement, and relationships) for the definition of the self, but focus on the interiority and spirituality, and a strong sense of connectedness with past and future. They argued that wisdom is a synthesis of the results of the three development stages: self-knowledge, detachment, and integration. This theory also uses a self-reporting scale to measure wisdom – adult self-transcendence inventory (ASTI; Levenson et al., 2005). Empirical studies using this scale also did not find significant gender effects on overall wisdom (Le and Levenson, 2005; Levenson et al., 2005; König and Glück, 2014).

Grossmann et al. (2010) argued that wisdom is a trait that helps people make wise decisions and involves wise reasoning that guides people through important challenges in social life. Another corresponding scale – situated wisdom reasoning scale (SWIS) – includes five dimensions: intellectual humility, recognition of multiple ways a situation may unfold and change, adopting an outsider viewpoint, recognition of perspectives of others, consideration of conflict resolution, and search for compromise (Brienza et al., 2018). Empirical research using this scale in a Chinese sample did not find any significant gender effect on overall wisdom (Wei and Wang, 2020).

Finally, Thomas et al. (2019a), based on theoretical models in psychology and neurobiology, developed the San Diego wisdom scale (SD-WISE) through a neurobiology lens; similarly, no significant gender differences in overall wisdom were found using SD-WISE (Lee et al., 2019).

#### Insignificant Gender Effect on the Levels of Wisdom Dimensions

Similarly, extensive studies have found no significant gender differences in the level of wisdom dimensions. These studies used various scales, including but not limited to 3D-WS (Alhosseini and Ferrari, 2019) and WDS (Wisdom Development Scale, Saleem et al., 2017), and both found that no gender effect on the level of wisdom dimensions was established.

In conclusion, the above studies indicated that there is no significant gender effect in overall wisdom or its dimensions. The scales used in these studies have a solid theoretical basis and ideal reliability and validity; therefore, these empirical results are reliable and accurate (Bangen et al., 2013). The empirical evidence supporting this hypothesis is presented in Table 1. Further, notably, due to the file-drawer problem (publication biases; Rosenthal, 1979), some papers that have not found significant gender effects of wisdom level may not be published and thus could not be retrieved and included in this analysis.

### Significant Gender Effect on Wisdom Level

#### Significant Gender Effect on the Levels of Wisdom Dimensions

**FIGURE 2:**
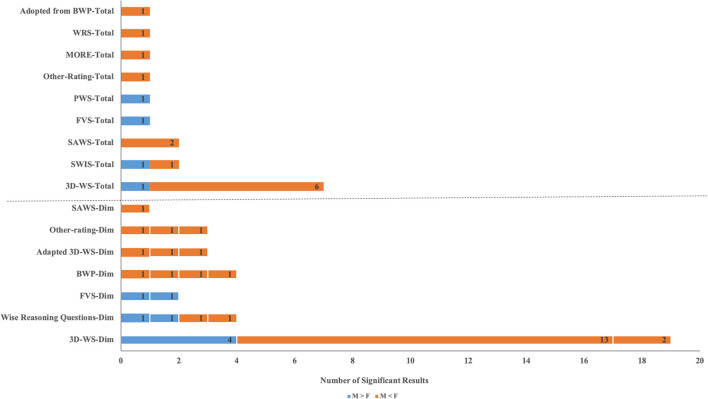
Summary of significant gender effects on levels of wisdom. Significant gender effects on levels of overall wisdom were shown above the horizontal dotted line, and significant gender effects on levels of wisdom dimensions were shown below the horizontal dotted line, with white vertical lines used to separate the significant results for the different dimensions of each scale. The names of dimensions with significant gender effects were as follows, in order from top to bottom and left to right: (1) SAWS: reminiscence; (2) Other rating: flexibility, perspective taking, recognition of limits of knowledge; (3) Adapted 3D-WS: nondualistic thinking, non-resentment, empathy; (4) BWP: use of procedural knowledge, lifespan perspective, relativism of values, management of uncertainty; (5) FVS: warmth*, intelligence*; (6) Wise reasoning questions: perspectives*, limits of knowledge*, emotional reactivity, compromise; (7) 3D-WS-Dim: reflective*, affective (compassionate), cognitive.

The difference between genders is mainly reflected in the affective dimension of wisdom (as shown in Table 2 and Figure 2). Using 3D-WS, researchers found that women scored significantly higher than men on the affective dimension (Cheraghi et al., 2015; Brudek and Sekowski, 2019). This dimension refers to showing compassion, benevolence, positive emotions and behavior toward people, and the motivation to promote their wellbeing. The typically measured item is “If I see people in need, I try to help them one way or another” (Ardelt, 2003). Thus, the higher scores of women on this dimension indicate that in the process of interpersonal communication, women are more sensitive to the emotions of others than men and have more positive behavior and emotional reaction, and fewer indifferent attitudes and negative reactions (García-Campayo et al., 2018). This gender difference was true both for individuals in early adulthood (18–29 years old; Beaumont, 2011) and the elderly (52–87 years old; Ardelt, 2009). Related to this, when recalling challenging life events, wise women were more sensitive than wise men and expressed more gratitude for the difficulties and those people who helped them (König and Glück, 2012). These findings are consistent with the widely held view that women are emotional animals (Eagly et al., 2019).

Gender differences in wisdom level are also seen in the reflective dimension (as shown in Table 2 and Figure 2). Webster (2003) recruited 217 early adults and found that women scored significantly higher than men on the reminiscence and reflectiveness dimension using SAWS. This suggests that women are more adept at recalling and reflecting on their life experiences. They try to gain life insights from these events and actively project them into a future life. However, Cheraghi et al. (2015) obtained different results using 3D-WS. Specifically, they found that there were no significant differences between young men and women (aged 18–35 years) in the reflective dimension of 3D-WS. As age increased, the reflective level of women showed a significant downward trend, while that of men was not obvious; therefore, the reflection score of older men was significantly higher than that of older women (over 55 years old). The reason for this contradictory result may be that, although the reflective dimensions of SAWS and 3D-WS are essentially the same, they show slight differences in detail. In other words, the reflective dimension in SAWS refers to the review and summary of experience of self, intending to learn from it and accumulate practical experience to better cope with future challenges. This belongs to the “wisdom about the self” category (Verhaeghen, 2019). The reflective dimension of 3D-WS refers to the examination of an individual of his/her own cognitive flexibility and objectivity. People with high scores in this dimension can effectively and flexibly adjust their thinking modes. This belongs to the “wisdom about the social world” category (Verhaeghen, 2019).

In addition, there were significant differences in interpersonal conflict coping styles between men and women (as shown in Table 2 and Figure 2). When in conflict with others, women were more likely than men to think from the perspective of others (i.e., perspective taking) and were more accurate in recognizing the limitations of their own knowledge, experiences, and abilities (Grossmann et al., 2012; Booker and Dunsmore, 2016). Rather than shelving conflicts or intensifying conflicts, women were more inclined to solve conflicts by integrating opinions from various parties or seeking compromise (Huynh et al., 2017). Additionally, in women, wise reasoning behavior in interpersonal conflict is not affected by the gender of the other party, whereas coping styles of men vary with the gender of the other party. When the counterparty was a woman, men used more wise reasoning strategies than when it was a man (Brienza et al., 2018).

#### Significant Gender Effect on the Levels of Overall Wisdom

Meanwhile, some studies have found significant gender effects on overall wisdom. These studies were based on different theories and measurement tools, but all found significant differences in the overall wisdom levels between men and women, as detailed in Table 2.

#### Possible Explanations for Gender Differences in Wisdom Levels

The gender differences in wisdom levels can be explained from the following three perspectives. The first is the biological perspective. Previous studies have found that genes, brain structure and function, and other physiological bases have important effects on psychological development and maturity (Halldorsdottir and Binder, 2017; Thomas et al., 2019b); there are considerable differences between genders in these physiological variables (Gershoni and Pietrokovski, 2017; Giddens et al., 2018, p. 285; Ristori et al., 2020). Thus, it is reasonable to believe that the gender effect on wisdom level is partly influenced by these physiological differences between genders. Another strong reason for this hypothesis is that the gender effects of various wisdom dimensions have been confirmed to be closely correlated with the physiological differences between genders.

The second perspective is the social division of labor and the socialization perspective. It argues that the physiological basis certainly affects wisdom level, but the influence of social and cultural factors is more important and is the primary factor leading to gender differences in wisdom level (Giddens et al., 2018, p. 288).

From the perspective of the social division of labor, although the situation has changed, in almost all cultures, men still occupy the instrumental role, and women occupy the expressive role (Giddens et al., 2018, p. 293). In other words, men pursue effective solutions to practical problems, whereas women value interpersonal interaction and emotional communication; men actively seek benefits, whereas women tend to avoid harm (Eagly et al., 2019). Therefore, when in conflict with others, men tend to choose more direct, intense communication and processing methods; women, however, generally try to consider the thoughts of the other person as much as possible to maintain a harmonious interpersonal relationship.

According to the gender socialization theory, the different social expectations of the two genders continue to influence and shape the actual behaviors of men and women in the socialization process, resulting in gender differences in real life (Giddens et al., 2018, pp. 283–284). In this process, to avoid negative evaluations or obtain more positive evaluations, individuals consciously or unconsciously pay attention to the gender-specific behavioral expectations commensurate with their biological sex and internalize these expectations into their own beliefs and codes of conduct (Giddens et al., 2018, pp. 283–284). Thus, these gender expectations transform into behavioral differences between the two genders. Specifically, women are relatively more communal and attach more importance to interpersonal relationships, emotional communication, and spirituality. Men, however, are more agentic, focus more on their own task performance in the group, and are willing to make scientific evaluations based on logic and empirical evidence (Ellemers, 2018). In the process of socialization, these gender expectations turn into the actual differences between men and women in their behavior performance, which is reflected in the empirical results of wisdom level: women score higher than men in the affective and reflective dimensions and avoid straight conflicts with others.

Role model learning is an important part of gender socialization. According to social learning theory, many behaviors of human beings are acquired through imitation, and the probability of same-sex imitation is much higher than that of opposite-sex imitation. Imitation of men role models by men and imitation of women role models by women are most likely to be appreciated by others and thus reinforced (Bussey and Bandura, 1999). Thus, individuals gradually learn the different expectations of genders in current social and cultural backgrounds, which provides the basis for the subsequent internalization process.

Third, there is an integrated perspective. It advocates that biological and social factors interact with each other to influence the wisdom level of an individual, leading to the generation of gender differences at the wisdom level. That is, social factors cannot affect individual cognition and personality development independently of biological factors, and similarly, the influence of innate physiological differences between men and women on wisdom level is regulated by acquired social and cultural factors (McCarthy, 2015; Lee and Jeste, 2019). Although the biological basis determines that there are great differences between men and women in some psychological variables, these natural differences may be reduced or even reversed after continuous social practice (Wood and Eagly, 2002).

#### Effect Sizes for Gender Differences in Wisdom Levels

Although some empirical studies have found a significant relationship between overall wisdom and gender (Ardelt and Jeste, 2018; Webster et al., 2018) and significant differences in wisdom levels between men and women (Cheraghi et al., 2015; Huynh et al., 2017), most had a small effect size: the absolute value of *r* ranges from 0.062 to 0.291; the absolute value range of partial *n*^2^ is 0.01 to 0.03. According to the judgment criteria for the magnitude of effect size proposed by Cohen (1988), these results were not ideal, or their explanatory powers were limited (see Table 2 for further details).

Similarly, the abovementioned significant gender differences in the levels of wisdom dimensions also had a small effect size, with Cohen’s *d* values less than 0.3, the absolute value range of *r* is 0.12–0.26, and the absolute value range of partial *n*^2^ is 0.011–0.04 (see Table 2 for further details).

Thus, it can be concluded that although some empirical studies have obtained significant gender effects on the level of overall wisdom, as well as wisdom dimensions, these effects were less obvious.

### Conclusion

For gender effects on the level of overall wisdom and its dimensions, although some studies have obtained significant results, these results have relatively small effect sizes. Thus, we can conclude that although differences exist between genders in the level of overall wisdom as well as affection, reflection, and ways of dealing with interpersonal conflict, these differences are not obvious. The gender similarity on most of the dimensions of wisdom can be proved by a meta-analysis research conducted by Hyde (2005). He reviewed 46 meta-analyses, each based on dozens of studies. In these 46 meta-analyses, except for 11 meta-analyses, all the other 35 meta-analyses can be regarded as wisdom components or dimensions. Results demonstrated that most of these studies did not find significant gender differences.

Before the systematic research of wisdom used empirical psychological approaches, people had a masculine tendency to define wisdom. For example, they believed that being more rational and wiser (view of Plato) were often regarded as common characteristics of men rather than that of women (Broverman et al., 1972; Gilligan, 2016). However, as more people recognize gender equality, this bias among researchers is gradually fading, and they are beginning to examine and explore wisdom more objectively. The findings of this research on the gender effect on wisdom level can confirm this to some extent.

Interestingly, as can be seen intuitively from Figure 2, the number of studies in which the wisdom level of women exceeds the wisdom level of men is more than the number of studies in which the wisdom level of men exceeds the wisdom level of women. Upon closer inspection, it is apparent that most of these results were obtained using the 3D-WS scale, and mostly, significant differences between genders were obtained in the affective dimension of this scale. It is well understood that women have a significant advantage over men in this dimension, which is consistent with the widely held view that women are more emotional in nature (Eagly et al., 2019). It is thus speculated that this phenomenon may be caused by the fact that the affective dimension in this commonly used wisdom scale carries a significant advantage for women.

## Future Direction

### Combination of Multiple Wisdom Measurements

Most of the abovementioned studies use self-reported or performance-based measures to explore the gender effect of wisdom levels; studies using case analysis (e.g., Yang, 2016) or literature analysis (e.g., Kim et al., 2020) were rarely mentioned in this research, as most of them rarely involve gender comparison and provide results on the gender effect on wisdom. Although the results were in line with the opinions of the general public, they are not rigorous (Jeste and Lee, 2019), as both assessment tools have shortcomings that cannot be ignored. Specifically, the self-reported wisdom scale is inevitably influenced by social desirability, individual subjective consciousness, and memory bias; all these elements negatively affect the objectivity and accuracy of assessment tools (Freund and Kasten, 2012). For example, responses of participants to items such as “I always try to look at all sides of a problem” (from 3D-WS) and “Emotions do not overwhelm me when I make personal decisions” (from SAWS) were easily affected by individual self-cognition.

Existing performance-based measures tend to equate wisdom with superior cognitive ability (Baltes and Staudinger, 2000) and problem-solving ability (Grossmann, 2012), overemphasize the position of the inner cognitive process in wisdom, and lack evaluation of the social value of problem-solving solutions. In other words, they ignore the investigation of “good morality,” which is a crucial component of wisdom.

Of course, we do not intend to deny all the existing wisdom assessment tools, but we simply remind researchers that the results obtained by a single measurement tool of wisdom may have large biases and, thus, cannot be used as the only basis for drawing conclusions. Therefore, in the future, in addition to perfecting the existing wisdom measurement, the organic integration of multiple assessment tools should be achieved as much as possible to obtain more targeted and convincing results. Examples of this kind of integration are a combination of wisdom self-rated scales and other-rated scales and that of self-reported or performance-based measures of wisdom.

### Strengthen Research on the Psychological Gender Effect of Wisdom

As discussed earlier, this research mostly focuses on the physiological sex effect of wisdom, and less attention is paid to the psychological gender effect. In fact, in terms of these two research orientations, the theoretical value and practical significance of the latter are more prominent. Psychological gender is significantly affected by social and cultural environments and thus has strong plasticity and variability (Cretella et al., 2019). Conversely, physiological sex is an innate biological characteristic of individuals that cannot be changed under normal circumstances. Therefore, even if the physical sex effect of wisdom is established, people can only passively accept this result. However, if the psychological gender effect of wisdom and its sub-components is established, then when conducting wisdom education and wisdom management, we can promote the generation and development of individual wisdom by shaping individual psychological gender.

### Focus on the Moderating Role of Age on the Relationship Between Wisdom and Gender

According to the intersectionality viewpoint in the field of gender psychology (Giddens et al., 2018, p. 283), gender is not a single demographic variable affecting individual psychology, and its focus only on gender in empirical studies is considerably limited. Specific to wisdom, in addition to gender, individual age also has an important impact on wisdom.

The main effects of gender and age on wisdom have been extensively investigated in existing separate studies, but little attention has been paid to the interaction between the two. Currently, only a few studies have explored this interaction. Cheraghi et al. (2015) divided the subjects into three age groups (18–34 years old as the young group, 35–54 years old as the middle age group, and over 55 years old as the elderly group) and used the 3D-WS scale as a tool to explore whether age can regulate the correlation between gender and wisdom level. The results showed that the scores of cognitive and reflective dimensions of women were significantly lower than that of men in the elderly group after controlling the education level of the participants. Whereas, in the youth group, the score of overall wisdom and affective dimension of women was significantly higher than that of men. Another study using 3D-WS found that for participants with intermediate education, the peak of wisdom of men occurred at the age of 45, while that of women occurred at the age of 61 (Ardelt et al., 2018). Therefore, it can be preliminarily speculated that the gender effects of wisdom and its dimensions vary in different age groups. Notably, both studies used 3D-WS as the assessment tool; therefore, it remains to be further explored whether other measurement tools can obtain similar significant results. Future research can adopt diversified scales and performance-based measures as well as horizontal comparisons and longitudinal follow-up studies to explore the interactive effects of gender and age more comprehensively on wisdom.

In addition to age, factors such as the education level (Ardelt et al., 2018) and cultural background (Brienza et al., 2018) of the individual may also affect the relationship between wisdom and gender. Future research can also expand the research on these topics by focusing on the interaction of these factors with wisdom.

## Conclusion

Based on the above untangling and summarizing, the following conclusions can be drawn. First, there are no significant differences in views of wisdom between men and women, nor in views of wise men and wise women between individuals. The former refers to the fact that men and women understand the connotations of wisdom similarly and have almost identical opinions of its real-life manifestations; this could be the result of the intra-cultural and group comparisons in most of the studies. The latter refers to the fact that in the eyes of the general public and professional researchers, wise men and women are extremely similar with high level of masculinity and femininity.

Second, although some empirical studies have obtained significant gender effects on overall wisdom, affective and reflective dimensions, and interpersonal conflict coping styles, the effect sizes of these significant results are relatively small. Thus, it can be said that this effect is less obvious.

In view of this conclusion, the rational attitude toward men and women should be to recognize women ideologically and acknowledge that women have almost the same competence, virtue, and independent personality as men. In production and life activities, recognizing the existence of gender differences allows both to freely develop their personal talents and make their own choices. Simultaneously, institutionally speaking, various measures should be adopted to give gender equality in legal, political, and economic opportunities and equal opportunities in remuneration for work and access to production resources. These include formulating gender-specific public policies, organizational strategies, and rules and regulations according to the different psychological and behavioral characteristics of men and women.

## Author Contributions

MX conceived and designed the study and wrote the manuscript. FW conceived the original idea, was in charge of overall direction and planning, and reviewed and edited the manuscript. Both authors contributed to the article and approved the submitted version.

## Conflict of Interest

The authors declare that the research was conducted in the absence of any commercial or financial relationships that could be construed as a potential conflict of interest.

## Publisher’s Note

All claims expressed in this article are solely those of the authors and do not necessarily represent those of their affiliated organizations, or those of the publisher, the editors and the reviewers. Any product that may be evaluated in this article, or claim that may be made by its manufacturer, is not guaranteed or endorsed by the publisher.

## References

[B1] Alhosseini F. Ferrari M. (2019). Effects of causal attribution and implicit mind-set on wisdom development. *Int. J. Aging Hum. Dev.* 90 319–336. 10.1177/0091415019836098 30887826

[B2] Ardelt M. (2003). Empirical assessment of a three-dimensional wisdom scale. *Res. Aging* 25 275–324. 10.1177/0164027503025003004

[B3] Ardelt M. (2009). How similar are wise men and women? A comparison across two age cohorts. *Res. Hum. Dev.* 6 9–26. 10.1080/15427600902779354

[B4] Ardelt M. Bruya B. (2020). Three-dimensional wisdom and perceived stress among college students. *J. Adult Dev.* 20 93–105. 10.1007/s10804-020-09358-w

[B5] Ardelt M. Ferrari M. (2019). Effects of wisdom and religiosity on subjective well-being in old age and young adulthood: Exploring the pathways through mastery and purpose in life. *Int. Psychogeriat.* 31 477–489. 10.1017/S1041610218001680 30457081

[B6] Ardelt M. Jeste D. V. (2018). Wisdom and hard times: The ameliorating effect of wisdom on the negative association between adverse life events and well-being. *J. Gerontol. B Psychol. Sci. Soc. Sci.* 73 1374–1383. 10.1093/geronb/gbw137 28329810 PMC6178964

[B7] Ardelt M. Pridgen S. Nutter-Pridgen K. L. (2018). The relation between age and three-dimensional wisdom: Variations by wisdom dimensions and education. *J. Gerontol. B Psychol. Sci. Soc. Sci.* 73 1339–1349. 10.1093/geronb/gbx182 29401232

[B8] Auer-Spath I. Glück J. (2019). Respect, attentiveness, and growth: Wisdom and beliefs about good relationships. *Int. Psychogeriat.* 31 1809–1821. 10.1017/S104161021900022X 30973127 PMC6925594

[B9] Baltes P. B. Staudinger U. M. (2000). Wisdom: A metaheuristic (pragmatic) to orchestrate mind and virtue toward excellence. *Am. Psychol.* 55 122–136. 10.1037/0003-066X.55.1.122 11392856

[B10] Bang H. Montgomery D. (2013). Wisdom and ego-identity for Korean and American late adolescents. *J. Cross-Cult. Psychol.* 44 807–813. 10.1177/0022022112466941

[B11] Bang H. Zhou Y. (2014). The function of wisdom dimensions in ego-identity development among Chinese university students. *Int. J. Psychol.* 49 434–445. 10.1002/ijop.12065 25355666

[B12] Bangen K. J. Meeks T. W. Jeste D. V. (2013). Defining and assessing wisdom: A review of the literature. *Am. J. Geriatric Psychiat.* 21 1254–1266. 10.1016/j.jagp.2012.11.020 23597933 PMC3896261

[B13] Beaumont S. L. (2011). Identity styles and wisdom during emerging adulthood: Relationships with mindfulness and savoring. *Identity* 11 155–180. 10.1080/15283488.2011.557298

[B14] Bergsma A. Ardelt M. (2012). Self-reported wisdom and happiness: An empirical investigation. *J. Happiness Stud.* 13 481–499. 10.1007/S10902-011-9275-5

[B15] Booker J. A. Dunsmore J. C. (2016). Profiles of wisdom among emerging adults: Associations with empathy, gratitude, and forgiveness. *J. Positive Psychol.* 11 315–325. 10.1080/17439760.2015.1081970

[B16] Brienza J. P. Kung F. Santos H. C. Bobocel D. R. Grossmann I. (2018). Wisdom, bias, and balance: Toward a process-sensitive measurement of wisdom-related cognition. *J. Personal. Soc. Psychol.* 115 1093–1126. 10.1037/pspp0000171 28933874

[B17] Broverman I. Vogel S. Broverman D. Clarkson F. Rosenkrantz P. (1972). Sex-Role stereotypes: A current appraisal. *J. Soc. Issues* 28 59–78. 10.1111/j.1540-4560.1972.tb00018.x

[B18] Brown S. C. Greene J. A. (2006). The wisdom development scale: Translating the conceptual to the concrete. *J. College Stud. Dev.* 47 1–19. 10.1353/csd.2006.0002 34409987

[B19] Brudek P. Sekowski M. (2019). Wisdom as the mediator in the relationships between meaning in life and attitude toward death. *Omega* 83 3–32. 10.1177/0030222819837778 30971185

[B20] Bruya B. Ardelt M. (2018). Wisdom can be taught: A proof-of-concept study for fostering wisdom in the classroom. *Learn. Instruc.* 58 106–114. 10.1016/J.LEARNINSTRUC.2018.05.001

[B21] Bushlack T. J. Bock T. (2018). Validating the “centering for wisdom assessment”: Assessing the role of contemplative practices in the cultivation of practical wisdom. *J. Psychol. Theol.* 46 143–167. 10.1177/0091647118764956

[B22] Bussey K. Bandura A. (1999). Social cognitive theory of gender development and differentiation. *Psychol. Rev.* 106 676–713. 10.1037/0033-295X.106.4.676 10560326

[B23] Cheraghi F. Kadivar P. Ardelt M. Asgari A. Farzad V. (2015). Gender as a moderator of the relation between age cohort and three-dimensional wisdom in Iranian culture. *Int. J. Aging Hum. Dev.* 81 3–26. 10.1177/0091415015616394 26610721

[B24] Cheung C.-K. Chow E. (2019). Contribution of wisdom to well-being in Chinese older adults. *Appl. Res. Qual. Life* 15 913–930. 10.1007/S11482-019-9712-X

[B25] Clayton V. Birren J. E. (1980). “The development of wisdom across the life span: A reexamination of an ancient topic,” in *Life-span development and behavior*, Vol. 3 eds Baltes P. B. Brim O. G. Jr. (Cambridge: Academic Press), 103–135.

[B26] Cohen J. (1988). *Statistical power analysis for the behavioral sciences*, 2nd Edn. Mahwah, NJ: Erlbaum.

[B27] Cretella M. A. Rosik C. H. Howsepian A. A. (2019). Sex and gender are distinct variables critical to health: Comment on Hyde, Bigler, Joel, Tate, and van Anders. *Am. Psychol.* 74 842–844. 10.1037/amp0000524 31580112

[B28] Dumbravă I. L. (2017). An empirical approach to wisdom processes. *Romanian J. Appl. Psychol.* 19 50–58. 10.24913/RJAP.19.2.04

[B29] Eagly A. H. Nater C. Miller D. I. Kaufmann M. Sczesny S. (2019). Gender stereotypes have changed: A cross-temporal meta-analysis of U.S. public opinion polls from 1946 to 2018. *Am. Psychol.* 75 301–315. 10.1037/amp0000494 31318237

[B30] Ellemers N. (2018). Gender stereotypes. *Annu. Rev. Psychol.* 69:275. 10.1146/annurev-psych-122216-011719 28961059

[B31] Ferrari M. Bang H. Ardelt M. Feng Z. (2019). Educating for virtue: How wisdom coordinates informal, non-formal and formal education in motivation to virtue in Canada and South Korea. *J. Moral Educ.* 48 47–64. 10.1080/03057240.2018.1546169

[B32] Freund P. A. Kasten N. (2012). How smart do you think you are? A meta-analysis on the validity of self-estimates of cognitive ability. *Psychol. Bull.* 138 296–321. 10.1037/a0026556 22181852

[B33] García-Campayo J. Del Hoyo Y. L. Barceló-Soler A. Navarro-Gil M. Borao L. Giarin V. (2018). Exploring the wisdom structure: Validation of the Spanish new short three-dimensional wisdom scale (3D-WS) and its explanatory power on psychological health-related variables. *Front. Psychol.* 9:692. 10.3389/fpsyg.2018.00692 29867662 PMC5960699

[B34] Gershoni M. Pietrokovski S. (2017). The landscape of sex-differential transcriptome and its consequent selection in human adults. *BioMed. Central Biol.* 15:7. 10.1186/s12915-017-0352-z 28173793 PMC5297171

[B35] Giddens A. Duneier M. Appelbaum R. P. Carr D. (2018). *Introduction to sociology*, 11th Edn. New York: W. W. Norton & Company.

[B36] Gilligan C. (2016). *In a different voice: Psychological theory and women’s development.* Cambridge: Harvard University Press.

[B37] Glück J. Bischof B. Siebenhüner L. (2012). “Knows what is good and bad”, “Can teach you things”, “Does lots of crosswords”: Children’s knowledge about wisdom. *Eur. J. Dev. Psychol.* 9 582–598. 10.1080/17405629.2011.631376

[B38] Glück J. Bluck S. Weststrate N. M. (2019). More on the MORE life experience model: What we have learned (so far). *J. Value Inquiry* 53 349–370. 10.1007/s10790-018-9661-x 31798190 PMC6887551

[B39] Glück J. König S. Naschenweng K. Redzanowski U. Dorner L. Straßer I. (2013). How to measure wisdom: Content, reliability, and validity of five measures. *Front. Psychol.* 4:405. 10.3389/fpsyg.2013.00405 23874310 PMC3709094

[B40] Glück J. Strasser I. Bluck S. (2009). Gender differences in implicit theories of wisdom. *Res. Hum. Dev.* 6 27–44. 10.1080/15427600902779370

[B41] Gordon J. K. Jordan L. M. (2017). Older is wiser? It depends who you ask and how you ask. *Aging Neuropsychol. Cogn.* 24 94–114. 10.1080/13825585.2016.1171292 27070433

[B42] Grossmann I. (2012). *Getting wisdom: Aging, culture and perspective* [Unpublished doctoral dissertation]. Ann Arbor, MI: University of Michigan.

[B43] Grossmann I. Karasawa M. Izumi S. Na J. Varnum M. E. W. Kitayama S. (2012). Aging and wisdom: Culture matters. *Psychol. Sci.* 23 1059–1066. 10.1177/0956797612446025 22933459

[B44] Grossmann I. Kross E. (2014). Exploring Solomon’s paradox: Self-distancing eliminates the self-other asymmetry in wise reasoning about close relationships in younger and older adults. *Psychol. Sci.* 25 1571–1580. 10.1177/0956797614535400 24916084

[B45] Grossmann I. Kung F. Y. H. (2018). “Wisdom and culture,” in *Handbook of cultural psychology*, 2nd Edn, eds Kitayama S. Cohen D. (New York: Guilford Press), 343–373.

[B46] Grossmann I. Na J. Varnum M. Park D. C. Kitayama S. Nisbett R. E. (2010). Reasoning about social conflicts improves into old age. *Proc. Natl. Acad. Sci.* 107 7246–7250. 10.1073/pnas.1001715107 20368436 PMC2867718

[B47] Grossmann I. Weststrate N. M. Ferrari M. Brienza J. P. (2020). A common model is essential for a cumulative science of wisdom. *Psychol. Inquiry* 31 185–194. 10.1080/1047840X.2020.1750920

[B48] Halldorsdottir T. Binder E. B. (2017). Gene × environment interactions: From molecular mechanisms to behavior. *Annu. Rev. Psychol.* 68:215. 10.1146/annurev-psych-010416-044053 27732803

[B49] Hartman P. S. (2000). *Women developing wisdom: Antecedents and correlates in a longitudinal sample* [Unpublished doctoral dissertation]. Ann Arbor, MI: University of Michigan.

[B50] Helgeson V. S. (2017). *Psychology of gender*, 5th Edn. Milton Park: Routledge.

[B51] Hu C. S. Ferrari M. Liu R. Gao Q. Weare E. (2018). Mainland Chinese implicit theory of wisdom: Generational and cultural differences. *J. Gerontol. B* 73 1416–1424. 10.1093/geronb/gbw157 27927747

[B52] Huynh A. C. Oakes H. Shay G. R. McGregor I. (2017). The wisdom in virtue: Pursuit of virtue predicts wise reasoning about personal conflicts. *Psychol. Sci.* 28 1848–1856. 10.1177/0956797617722621 28972825

[B53] Hyde J. S. (2005). The gender similarities hypothesis. *Am. Psychol.* 60 581–592. 10.1037/0003-066X.60.6.581 16173891

[B54] Hyde J. S. Bigler R. S. Joel D. Tate C. C. Anders S. M. (2019). The future of sex and gender in psychology: Five challenges to the gender binary. *Am. Psychol.* 74 171–193. 10.1037/amp0000307 30024214

[B55] Jason L. A. Helgerson J. L. Torres-Harding S. Fries M. Carrico A. Chimata R. (2004). A scale to measure wisdom. *Hum. Psychol.* 32 284–305. 10.1080/08873267.2004.9961756

[B56] Jason L. A. Reichler A. King C. Madsen D. Camacho J. Marchese W. (2001). The measurement of wisdom: A preliminary effort. *J. Commun. Psychol.* 29 585–598. 10.1002/JCOP.1037

[B57] Jeste D. V. Lee E. E. (2019). The emerging empirical science of wisdom: Definition, measurement, neurobiology, longevity, and interventions. *Harvard Rev. Psychiat.* 27 127–140. 10.1097/HRP.0000000000000205 31082991 PMC6519134

[B58] Kim J. J. Feng Z. Ferrari M. (2020). Foresight and wisdom: The case of the classic of changes. *Hum. Psychol.* 2020:194. 10.1037/hum0000194

[B59] König S. Glück J. (2012). Situations in which I was wise: Autobiographical wisdom memories of children and adolescents. *J. Res. Adolesc.* 22 512–525. 10.1111/J.1532-7795.2012.00800.X

[B60] König S. Glück J. (2014). “Gratitude is with me all the time”: How gratitude relates to wisdom. *J. Gerontol. B Psychol. Sci. Soc. Sci.* 69 655–666. 10.1093/geronb/gbt123 24326079 PMC4141499

[B61] Kordacová J. (2010). Wisdom and irrationality: Probe into mutual relationships. *Stud. Psychol.* 52 339–346.

[B62] Krause N. (2016). Assessing the relationships among wisdom, humility, and life satisfaction. *J. Adult Dev.* 23 140–149. 10.1007/S10804-016-9230-0

[B63] Krause N. Hayward R. D. (2014). Religious involvement, practical wisdom, and self-rated health. *J. Aging Health* 26 540–558. 10.1177/0898264314524437 24619064

[B64] Krause N. Hayward R. D. (2015). Assessing whether practical wisdom and awe of God are associated with life satisfaction. *Psychol. Religion Spirit.* 7 51–59. 10.1037/A0037694

[B65] Kunzmann U. (2019). “Performance-based measures of wisdom: State of the art and future directions,” in *The Cambridge handbook of wisdom*, eds Sternberg R. J. Glück J. (Cambridge: Cambridge University Press), 277–296.

[B66] Le T. N. (2008). Age differences in spirituality, mystical experiences and wisdom. *Ageing Soc.* 28 383–411. 10.1017/S0144686X0700685X

[B67] Le T. N. Levenson M. R. (2005). Wisdom as self-transcendence: What’s love (& individualism) got to do with it? *J. Res. Personal.* 39 443–457. 10.1016/j.jrp.2004.05.003

[B68] Lee E. E. Depp C. Palmer B. W. Glorioso D. Daly R. Liu J. (2019). High prevalence and adverse health effects of loneliness in community-dwelling adults across the lifespan: Role of wisdom as a protective factor. *Int. Psychogeriat.* 31 1447–1462. 10.1017/S1041610218002120 30560747 PMC6581650

[B69] Lee E. E. Jeste D. V. (2019). “Neurobiology of wisdom,” in *The Cambridge handbook of wisdom*, eds Sternberg R. J. Glück J. (Cambridge: Cambridge University Press.), 69–94.

[B70] Levenson M. R. Jennings P. A. Aldwin C. M. Shiraishi R. W. (2005). Self-transcendence: Conceptualization and measurement. *Int. J. Aging Hum. Dev.* 60 127–143. 10.2190/XRXM-FYRA-7U0X-GRC0 15801386

[B71] McCarthy M. M. (2015). *Sex differences in the brain: How male and female brains diverge is a hotly debated topic, but the study of model organisms points to differences that cannot be ignored.* Delaware: The Scientist.

[B72] Moraitou D. Efklides A. (2012). The wise thinking and acting questionnaire: The cognitive facet of wisdom and its relation with memory, affect, and hope. *J. Happiness Stud.* 13 849–873. 10.1007/S10902-011-9295-1

[B73] Orwoll L. Achenbaum W. A. (1993). Gender and the development of wisdom. *Hum. Dev.* 36 274–296. 10.1159/000278214

[B74] Pasupathi M. Staudinger U. (2001). Do advanced moral reasoners also show wisdom? Linking moral reasoning and wisdom-related knowledge and judgement. *Int. J. Behav. Dev.* 25 401–415. 10.1080/016502501316934833

[B75] Plato. (1892). *The dialogues of Plato* (B. Jowett, Trans, Vol. 3. Oxford: Oxford University Press.

[B76] Plato. (1902/2009). *The republic of Plato*, Vol. 1. Cambridge: Cambridge University Press.

[B77] Ristori J. Cocchetti C. Romani A. Mazzoli F. Vignozzi L. Maggi M. (2020). Brain sex differences related to gender identity development: Genes or hormones? *Int. J. Mol. Sci.* 21:2123. 10.3390/ijms21062123 32204531 PMC7139786

[B78] Rosenthal R. (1979). The file drawer problem and tolerance for null results. *Psychol. Bull.* 86 638–641. 10.1037/0033-2909.86.3.638

[B79] Saleem G. Hasan S. S. Fayyaz W. (2017). Relationship of epistemological development with wisdom, age, gender and education. *Pakistan J. Soc. Clin. Psychol.* 15 27–35.

[B80] Sternberg R. J. (1990). “Wisdom and its relations to intelligence and creativity,” in *Wisdom: Its nature, origins, and development*, ed. Sternberg R. J. (Cambridge: Cambridge University Press), 142–159.

[B81] Takahashi M. Overton W. F. (2002). Wisdom: A culturally inclusive developmental perspective. *Int. J. Behav. Dev.* 26 269–277. 10.1080/01650250143000139

[B82] Thomas M. L. Bangen K. J. Palmer B. W. Sirkin Martin A. Avanzino J. A. Depp C. A. (2019a). A new scale for assessing wisdom based on common domains and a neurobiological model: The San Diego Wisdom Scale (SD-WISE). *J. Psychiat. Res.* 108 40–47. 10.1016/j.jpsychires.2017.09.005 28935171 PMC5843500

[B83] Thomas M. L. Martin A. S. Eyler L. Lee E. E. Jeste D. V. (2019b). Individual differences in level of wisdom are associated with brain activation during a moral decision-making task. *Brain Behav.* 9:e01302. 10.1002/brb3.1302 31044549 PMC6577614

[B84] Verhaeghen P. (2019). The examined life is wise living: The relationship between mindfulness, wisdom, and the moral foundations. *J. Adult Dev.* 27 1–18. 10.1007/s10804-019-09343-y

[B85] Wang F. Y. (2019). *Chinese culture psychology: A new look.* Shanghai: Shanghai Educational Publishing House.

[B86] Wang F. Y. Zheng H. (2014). *Theoretical exploration and applied researchers of wisdom psychology.* Shanghai: Shanghai Education Publishing House.

[B87] Wang Z. D. Wang Y. M. Li K. Shi J. Wang F. Y. (2021). The comparison of the wisdom view in Chinese and Western cultures. *Curr. Psychol.* 2021 1–12. 10.1007/s12144-020-01226-w 33424207 PMC7786156

[B88] Webster J. D. (2003). An exploratory analysis of a self-assessed wisdom scale. *J. Adult Dev.* 10 13–22. 10.1023/A:1020782619051

[B89] Webster J. D. (2007). Measuring the character strength of wisdom. *Int. J. Aging Hum. Dev.* 65 163–183. 10.2190/AG.65.2.D 17957986

[B90] Webster J. D. (2019). “Self-report wisdom measures: Strengths, limitations, and future directions,” in *The Cambridge handbook of wisdom*, eds Sternberg R. J. Glück J. (Cambridge: Cambridge University Press), 297–320.

[B91] Webster J. D. Bohlmeijer E. T. Westerhof G. J. (2014). Time to flourish: The relationship of temporal perspective to well-being and wisdom across adulthood. *Aging Mental Health* 18 1046–1056. 10.1080/13607863.2014.908458 24807401

[B92] Webster J. D. Weststrate N. M. Ferrari M. Munroe M. Pierce T. W. (2018). Wisdom and meaning in emerging adulthood. *Emerg. Adult.* 6 1–19. 10.1177/2167696817707662

[B93] Wei X. D. Wang F. Y. (2020). Southerners are wiser than Northerners regarding interpersonal conflicts in China. *Front. Psychol.* 11:225. 10.3389/fpsyg.2020.00225 32132958 PMC7040192

[B94] Weststrate N. M. Ferrari M. Ardelt M. (2016). The many faces of wisdom: An investigation of cultural-historical wisdom exemplars reveals practical, philosophical, and benevolent prototypes. *Personal. Soc. Psychol. Bull.* 42 662–676. 10.1177/0146167216638075 27052325

[B95] Wink P. Dillon M. (2003). Religiousness, spirituality, and psychosocial functioning in late adulthood: Findings from a longitudinal study. *Psychol. Aging* 18 916–924. 10.1037/0882-7974.18.4.916 14692876

[B96] Wink P. Staudinger U. M. (2016). Wisdom and psychosocial functioning in later life. *J. Personal.* 84 306–318. 10.1111/jopy.12160 25546500

[B97] Wood W. Eagly A. H. (2002). A cross-cultural analysis of the behavior of women and men: Implications for the origins of sex differences. *Psychol. Bull.* 128 699–727. 10.1037/0033-2909.128.5.699 12206191

[B98] Yang S. Y. (2008). Real-life contextual manifestations of wisdom. *Int. J. Aging Hum. Dev.* 67 273–303. 10.2190/AG.67.4.a 19266867

[B99] Yang S. Y. (2011). Wisdom displayed through leadership: Exploring leadership-related wisdom. *Leadership Q.* 22 616–632. 10.1016/J.LEAQUA.2011.05.004

[B100] Yang S. Y. (2016). Exploring wisdom in the Confucian tradition: Wisdom as manifested by Fan Zhongyan. *New Ideas Psychol.* 41 1–7. 10.1016/j.newideapsych.2015.11.001

[B101] Yang S. Y. Intezari A. (2019). “Non-Western lay conceptions of wisdom,” in *The Cambridge handbook of wisdom*, eds Sternberg R. J. Glück J. (Cambridge: Cambridge University Press), 429–452.

[B102] Yao X. Z. (2000). *An introduction to Confucianism.* Cambridge: Cambridge University Press.

[B103] Zacher H. Mckenna B. Rooney D. (2013). Effects of self-reported wisdom on happiness: Not much more than emotional intelligence? *J. Happiness Stud.* 14 1697–1716. 10.1007/S10902-012-9404-9

[B104] Zhu G. L. (2006). *Confucian ideal personality and Chinese culture.* Shanghai: Fudan University Press.

